# Supplementary material for: Solving multi-scenario hybrid flow shop scheduling problem based on an improved probe machine model
Source: PLoS One. 2025 Sep 3;20(9):e0330020. doi: 10.1371/journal.pone.0330020 (PMC12407458; doi:10.1371/journal.pone.0330020)
Supplement: Attachment — Submitted filename: Response to Reviewers.pdf [file pone.0330020.s002.pdf]

# Responses to "Reviewers' comments"

regarding to

"*Solving multi-scenario hybrid flow shop scheduling problem based on an improved probe machine model*"

Dear Reviewers and Editors,

Thank you very much for your useful comments and suggestions on the content of our manuscript. Your comments are very valuable and helpful for revising and improving our work. We have seriously considered each comment and modified the manuscript accordingly. The detailed corrections are listed below:

***Reviewer #1：***

1) How to reflect the multi-scenario hyrbid flow shop problem?

✓ Thanks for the comments.

We sincerely appreciate this insightful comment. The multi-scenario feature is fundamentally reflected in the following aspects:

**Unified data modeling:** The tuple set $M_{OId,j,pt}$ designed in Section 4.1 enables seamless handling of both HFS-IPM and HFS-UPM data. This means that a unified data input format can be adopted without altering the structure of the model. A screenshot of the relevant description in Section 4.1 is provided below.

Data preprocessing contributes to enhancing the interpretability of data, thereby providing a more reliable and effective foundation for subsequent modeling process. The data structure $M_{OId,j,pt}$ proposed in this work can be effectively compatible with both HFS-IPM and HFS-UPM data types. The tuples mentioned above, $< 1, 1, 24 >$, $< 1, 2, 75 >$, and $< 1, 3, 51 >$, serve as examples. They indicate that for operation 1, the optional machines are 1, 2, and 3, with corresponding processing times of 24, 75, and 51 respectively. It is evident that the processing time of the same operation on different optional machines is not the same, which constitutes an HFS-UPM problem. For the HFS-IPM problem, the possible tuples are in the form of $< 1, 1, 24 >$, $< 1, 2, 24 >$ and $< 1, 3, 24 >$. That is, the processing time of the same operation on different optional machines is the same. In summary, the data structure $M_{OId,j,pt}$ can satisfy the data feeding of these two types of HFS at the same time without affecting the construction of the model.

**Dynamic constraint switching:** The core CP model remains unchanged across different scenarios. Switching between standard HFS and no-wait HFS is achieved solely by modifying the corresponding spatiotemporal constraints (Eq. 44 and Eq. 48).

**Experimental coverage:** Tables 4–12 verify all four scenario combinations, and Figures 18–19 present Gantt charts as evidence based on real-world cases.

**Supplementary revisions in the main text:** To more clearly illustrate the four scenarios, we have added the following content in Section 4.2.

**Parallel machine scenario adaptability:** The unified data structure $M_{OId,j,pt}$ inherently supports both HFS-IPM and HFS-UPM scenarios. For HFS-IPM, processing times satisfy $\forall j_1, j_2 : pt(t, j_1) = pt(t, j_2)$; for HFS-UPM, $pt(t, j)$ varies arbitrarily. This design eliminates scenario-specific modeling.

**Scenario switching mechanism:** The transition between *standard* and *no-wait* scenarios requires only one constraint change: Replace `endBeforeStart` (Eq 44) with `endAtStart` (Eq 48), demonstrating extreme flexibility in multi-scenario adaptation.

Further, based on the interval variable $Itvs$ above, an interval sequence variable $Seq$ is defined to represent the possible ordering of this interval variable. The dedicated notations involved in the IPMMPO-CP model and their meanings are shown in Table 3.

Detailed changes are in the revised manuscript with track changes.

Please check.

2) Please provide mathematical model of the multi-scenario HFSP. Does multi-scenario HFSP have the same mathematical model or different mathematical models?

✓ Thanks for the comments. We sincerely appreciate this sharp observation.

The four HFS scenarios investigated in this study share a unified mathematical model. In the revised version, we have added a detailed description in Section 2.1, including Table 1 (notations and their definitions), Equations (1)–(10), and the corresponding explanations of these equations. A screenshot of the added content is provided below.

problem is to minimize the maximum completion time. The mathematical model of the HFS problem is described as follows (Eq 1 - 10), with the notations and their corresponding meanings presented in Table 1. Following the rules of [66], the HFS problems studied in this work can be denoted as $HF_s \parallel C_{\max}$.

**Table 1.** Dedicated notations and the corresponding meanings

| Notation | Meaning |
|---|---|
| $n$ | number of jobs. |
| $N$ | set of jobs, $N = \{1, 2, \cdots, n\}$. |
| $i$ | index of the job, $i \in N$. |
| $m$ | total number of machines. |
| $s$ | total number of stages. |
| $L$ | set of stages, $L = \{1, 2, \cdots, s\}$. |
| $k$ | index for stages, $k \in L$. |
| $m_k$ | the total number of available machines at stage $k$. |
| $M_k$ | the set of available machines at stage $k$. |
| $j$ | the index of available machines at stage $k$, $j \in M_k$. |
| $B$ | a sufficiently large positive number. |
| $p_{k,i}$ | the processing time of job $i$ at stage $k$ (given in advance). |
| $C_{k,i}$ | the completion time of job $i$ at stage $k$. |
| $u_{i,i',j,k}$ | decision variable, equal to 1 if jobs $i$ and $i'$ are both assigned to machine $j$ at stage $k$, and job $i$ is the immediate predecessor of job $i'$; 0 otherwise. |
| $C_{max}$ | the maximum completion time (makespan). |

$$MinC_{\max} = Min\left[\underset{i\in N}{Max}\, C_{s,i}\right] \tag{1}$$

Subject to:

$$\sum_{i=0,i\neq i'}^{n}\sum_{j=1}^{m_k} u_{i,i',j,k} = 1,\ \forall i' \in N, k \in L \tag{2}$$

$$\sum_{i'=0,i'\neq i}^{n}\sum_{j=1}^{m_k} u_{i,i',j,k} = 1,\ \forall i \in N, k \in L \tag{3}$$

$$\sum\nolimits_{i=0}^{n} u_{0,i,j,k} \leq 1,\ \forall k \in L, j \in M_k \tag{4}$$

$$\sum\nolimits_{i=0}^{n} u_{i,0,j,k} \leq 1,\ \forall k \in L, j \in M_k \tag{5}$$

$$\sum_{i=0,i\neq i'}^{n} u_{i',i,j,k} - \sum_{i=0,i\neq i'}^{n} u_{i,i',j,k} = 0,\ \forall i' \in N, k \in L, j \in M_k \tag{6}$$

$$C_{k,i} + p_{k,i'} \cdot \sum\nolimits_{j=1}^{m_k} u_{i,i',j,k} + B \cdot (\sum\nolimits_{j=1}^{m_k} u_{i,i',j,k} - 1) \leq C_{k,i'},\ \forall k \in L,\ i, i' \in N \tag{7}$$

$$C_{k,i} \geq C_{k-1,i} + p_{k,i},\ \forall k \in L, i \in N \tag{8}$$

$$C_{0,i} = 0,\ \forall i \in N \tag{9}$$

$$C_{s,i} \leq C_{\max},\ \forall i \in N \tag{10}$$

Constraints 2, 3 and 6 ensure that each job at every stage has exactly one immediate predecessor and one immediate successor. At the same time, they enforce that each job at every stage is processed by exactly one of the available machines. Constraints 4 and 5 ensure that, on each machine at every stage, exactly one job is processed first and exactly one job is processed last, respectively. Constraint 7 represents the time constraint between two jobs with a direct predecessor-successor relationship on the same machine at the same stage. Constraint 8 imposes the precedence constraint between different processing stages of the same job. Constraint 9 states that the earliest start time for any job is zero, indicating that there is no priority constraint on the initial processing of different jobs.

As mentioned in our response to the first comment, the design of the tuple set $M_{Old,j,pt}$ effectively addresses the data compatibility issue between HFS-IPM (processing time homogeneous) and HFS-UPM (processing time heterogeneous) scenarios. In addition, switching between standard HFS and no-wait HFS can be achieved by simply modifying a single constraint (see Eq. 44 and Eq. 48). This approach avoids the need for ad hoc modeling for each scenario and demonstrates the flexibility of our method in handling multi-scenario applicability.

Detailed changes are in the revised manuscript with track changes.

Please check.

3) In introduction, there are various studies which have a close relation with the topic considered in this paper, such as An automatic multi-objective evolutionary algorithm for the hybrid flowshop scheduling problem with consistent sublots, and Mathematical model and knowledge-based iterated greedy algorithm for distributed assembly hybrid flow shop scheduling problem with dual-resource constraints. The review of this paper will be further enriched if they are included.

✓ Thanks for the comments.

We sincerely appreciate your recommendation of the two important references that are highly relevant to our study. Your suggested literature precisely supplements the latest developments in this field and significantly enhances the completeness of the research background. Following your suggestion, we have incorporated the following content into the revised Introduction, specifically in the third paragraph on page 2 of the manuscript:

[Introduction of the revised manuscript, the third paragraph on page 2 of the manuscript:]

iterated local search [23], iterated greedy (IG) [9], etc. For the distributed assembly shop hybrid flow shop (HFS) problem with dual resource constraints, some scholars have proposed a mixed-integer linear programming (MILP) model and a knowledge-based iterative greedy (KBIG) algorithm, aiming to minimize the total tardiness [24]. For the HFS problem with consistent sublots and dual objectives of minimizing the makespan and the total number of sublots, Zhang et al. [25] formulated a MIP model and proposed an automatic algorithm design approach to solve it.

[References:]


24. Yu, Fei and Lu, Chao and Zhou, Jiajun and Yin, Lvjiang,“Mathematical model and knowledge-based iterated greedy algorithm for distributed assembly hybrid flow shop scheduling problem with dual-resource constraints,” *Expert Systems with Applications*, vol. 239, pp. 122434, 2024, doi:10.1016/j.eswa.2023.122434.
25. Zhang, Biao and Pan, Quan-ke and Meng, Lei-lei and Lu, Chao and Mou, Jian-hui and Li, Jun-qing,“An automatic multi-objective evolutionary algorithm for the hybrid flowshop scheduling problem with consistent sublots,” *Knowledge-Based Systems*, vol. 238, pp. 107819, 2022, doi:10.1016/j.knosys.2021.107819.


Detailed changes are in the revised manuscript with track changes.

Please check.

4) In the experiment, statistic test like t-test should be conducted to validate the significance difference between difference metaheuristics for large-scale problems due to the statistical probability characteristics of these metaheuristics. Moreover, the deep reason why the proposal is better than its counterparts should be stated more clearly in the result analysis.

✓ Thanks for the comments.

Your suggestion has made the experimental comparisons in our study more comprehensive and convincing. In Section

5.2, we have added the ANOVA results and Tukey HSD tests between our CP model and the three comparison algorithms on large-scale benchmark instances. The differences are statistically significant, as evidenced by both the p-values and the comparison between F and F_crit. Screenshots of the newly added Tables 7 and 8 are provided below, and the corresponding paragraph in the manuscript has also been revised accordingly with necessary explanations.

**Table 7.** ANOVA results from multiple repetitions on large-scale benchmark

| Source of Variation | SS | df | MS | F | P-value | F crit |
|---|---|---|---|---|---|---|
| Between Groups | 426.5346 | 3 | 142.1782 | 57.44706 | 8.43E-14 | 2.866266 |
| Within Groups | 89.09794 | 36 | 2.474943 | | | |
| Total | 515.6325 | 39 | | | | |

**Table 8.** Multiple comparison of means - Tukey HSD

| Group1 | Group2 | Meandiff | P-adj | lower | upper | reject |
|---|---|---|---|---|---|---|
| DABC | HEA | -5.4429 | 0.0000 | -7.3377 | -3.5481 | True |
| DABC | IDABC | -1.7901 | 0.0698 | -3.6849 | 0.1047 | False |
| DABC | our CP | -8.4401 | 0.0000 | -10.3349 | -6.5453 | True |
| HEA | IDABC | 3.6528 | 0.0000 | 1.758 | 5.5476 | True |
| HEA | our CP | -2.9972 | 0.0008 | -4.892 | -1.1024 | True |
| IDABC | our CP | -6.65 | 0.0000 | -8.5448 | -4.7552 | True |

In the original manuscript, Section 5 presents comparisons with nine approximation algorithms, while Section 6 focuses on comparisons with two exact algorithms. To more clearly elaborate on the underlying reasons why our approach outperforms the existing methods, we have added corresponding summary paragraphs at the end of both Section 5 and Section 6 in the revised manuscript. These additions aim to analyze the deeper reasons behind the superiority of our approach. Screenshots of the added content are provided below.

[The last paragraph of Section 5:]

In this section, we mainly compare our approach with nine representative approximation methods. The experimental results demonstrate that IPMMPO-CP outperforms the competing algorithms in both solution time and solution accuracy. This advantage is attributed to the CP model's use of efficient constraint propagation algorithms and domain reduction techniques as its search and inference engine, as well as its support for multi-core and multi-threaded parallel optimization. Not only that, CP methods can even prove that the best feasible solution found is optimal when the search automatically terminates [59,64].

[The last paragraph of Section 6:]

In this section, we mainly compare our approach with two recent and representative CP models, namely CP1 and CP2. The CP1 model lacks cumulative constraints, which may result in a less compact search space and consequently higher computational time. Although the CP2 model employs cumulative constraints, they are fundamentally different from those used in our study. As shown in Tables 11 - 12, the number of constraints in the CP2 model (see the "#Cstr" indicator) is 2 - 3 times greater than that of IPMMPO-CP, which significantly increases the model's complexity. In summary, in terms of solution time, solution accuracy, and model compactness, IPMMPO-CP outperforms both CP1 and CP2.

Detailed changes are in the revised manuscript with track changes.

Please check.

***Reviewer #2：***

1) Although the authors propose the IPMMPO-CP model and demonstrate its effectiveness on several instances, the experimental validation mainly focuses on specific case studies. There may be concerns about the model's generalizability and robustness across various real-world manufacturing scenarios.

✓ Thanks for the comments. We sincerely appreciate your valuable comments on our work. The issue you raised regarding model generalization and robustness is indeed of great importance.

**Scenario Robustness Design:** This study mainly investigates four types of HFS scenarios: HFS-IPM, HFS-UPM, standard HFS, and no-wait HFS. Among them, HFS-IPM and HFS-UPM depend on the type of parallel machines. As described in Section 4.1, the proposed data structure $M_{OId,j,pt}$ effectively unifies these two scenarios and ensures compatibility in data input. That is, after unifying the data structure, both HFS-IPM and HFS-UPM can be solved using the same IPMMPO-CP model. Additionally, the standard HFS and no-wait HFS scenarios can be converted into each other by simply modifying a single constraint (e.g., replacing Eq. 44 with Eq. 48), without any changes to the core model structure. As for other scenarios beyond the scope of this study, we will further investigate and address them in our future work. Screenshots of the revised content related to the design and description of the tuple structure $M_{OId,j,pt}$ in Section 4.1, as well as the additions regarding the four types of scenarios in Section 4.2, are presented below.

[the description of the tuple structure $M_{OId,j,pt}$ in Section 4.1:]

Data preprocessing contributes to enhancing the interpretability of data, thereby providing a more reliable and effective foundation for subsequent modeling process. The data structure $M_{OId,j,pt}$ proposed in this work can be effectively compatible with both HFS-IPM and HFS-UPM data types. The tuples mentioned above, $<1,1,24>$, $<1,2,75>$, and $<1,3,51>$, serve as examples. They indicate that for operation 1, the optional machines are 1, 2, and 3, with corresponding processing times of 24, 75, and 51 respectively. It is evident that the processing time of the same operation on different optional machines is not the same, which constitutes an HFS-UPM problem. For the

HFS-IPM problem, the possible tuples are in the form of $< 1, 1, 24 >$, $< 1, 2, 24 >$ and $< 1, 3, 24 >$. That is, the processing time of the same operation on different optional machines is the same. In summary, the data structure $M_{OId,j,pt}$ can satisfy the data feeding of these two types of HFS at the same time without affecting the construction of the model.

[Supplementary explanations on the four scenarios in Section 4.2:]

**Parallel machine scenario adaptability:** The unified data structure $M_{OId,j,pt}$ inherently supports both HFS-IPM and HFS-UPM scenarios. For HFS-IPM, processing times satisfy $\forall j_1, j_2 : pt(t, j_1) = pt(t, j_2)$; for HFS-UPM, $pt(t, j)$ varies arbitrarily. This design eliminates scenario-specific modeling.

**Scenario switching mechanism:** The transition between *standard* and *no-wait* scenarios requires only one constraint change: Replace `endBeforeStart` (Eq 44) with `endAtStart` (Eq 48), demonstrating extreme flexibility in multi-scenario adaptation.

Further, based on the interval variable $Itvs$ above, an interval sequence variable $Seq$ is defined to represent the possible ordering of this interval variable. The dedicated notations involved in the IPMMPO-CP model and their meanings are shown in Table 3.

**Validation on Real-World Industrial Cases:** In the application comparison presented in Section 6, *RealCase1* and *RealCase2* are two real-world industrial cases obtained from a water meter manufacturer. In particular, *RealCase2* is a large-scale case involving 50 jobs, 22 machines, and 6 stages. Tables 11 and 12 report the application comparison results under the standard HFS and no-wait HFS scenarios, respectively. In addition, Figures 18 and 19 illustrate the scheduling Gantt charts for the two scenarios, which further demonstrate the effectiveness of IPMMPO-CP in handling complex industrial scheduling problems.

**Table 11.** Comparison with CP2 [72] in the standard HFS scenario

| Instance | CP2 | | | IPMMPO-CP | | |
|---|---|---|---|---|---|---|
| | Best | #Cstr | CPU[a] | Best | #Cstr | CPU[a] |
| BenCase1 | **23** | 175 | 0.39 | **23** | 76 | 0.25 |
| BenCase2 | **297** | 212 | 0.23 | **297** | 102 | 0.18 |
| RealCase1 | **479** | 212 | 16.93 | **479** | 102 | 3.5 |
| RealCase2 | 1901 | 1660 | 489.26 | 1887 | 582 | 384.75 |
| Average | | | 126.70 | | | 97.17 |

[a] The first time the CP solver finds the optimal/best solution.

**Table 12.** Comparison with CP2 [72] in the no-wait HFS scenario

| Instance | CP2 | | | IPMMPO-CP | | |
|---|---|---|---|---|---|---|
| | Best | #Cstr | CPU[a] | Best | #Cstr | CPU[a] |
| BenCase1 | **23** | 175 | 1.49 | **23** | 76 | 0.3 |
| BenCase2 | **297** | 212 | 29.08 | **297** | 102 | 2.65 |
| RealCase1 | **485** | 212 | 7.65 | **485** | 102 | 1.72 |
| RealCase2 | 1966 | 1660 | 542.63 | 1955 | 582 | 487.36 |
| Average | | | 145.21 | | | 123.01 |

[a] The first time the CP solver finds the optimal/best solution.

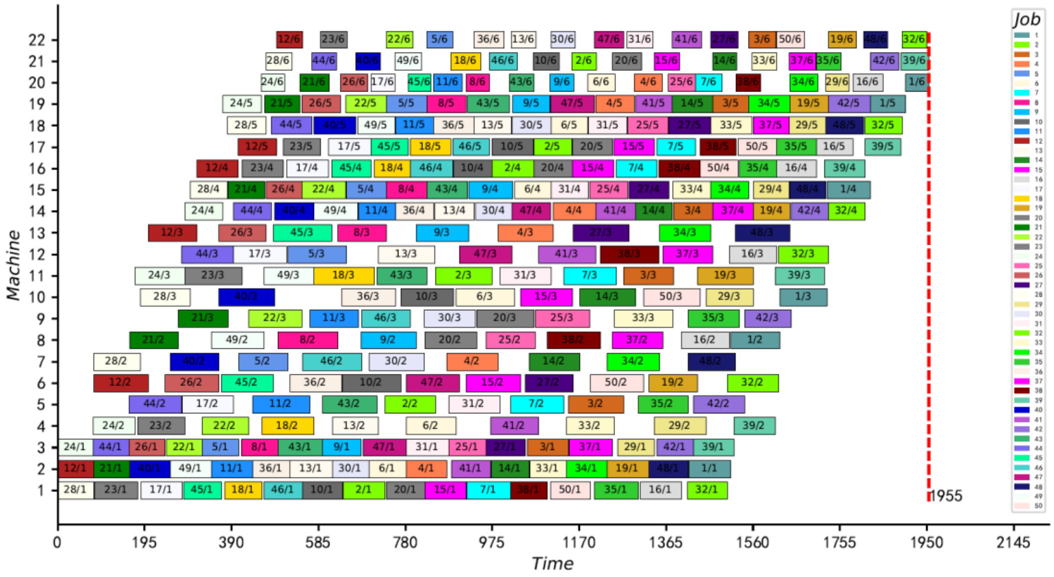


Fig 18. Gantt chart corresponding to the best feasible solution of RealCase2 in the no-wait HFS scenario.

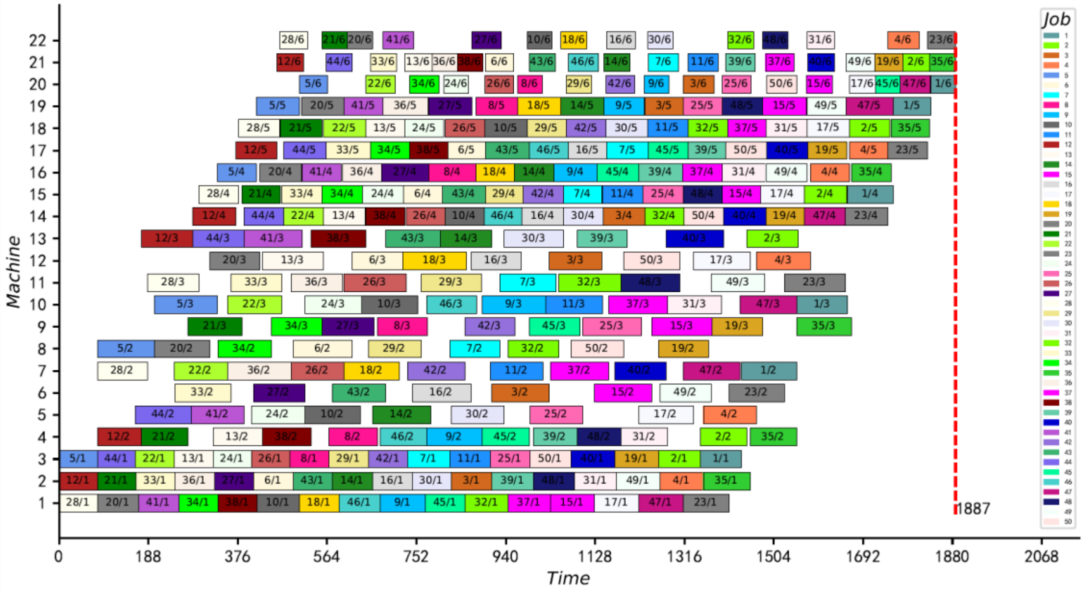


Fig 19. Gantt chart corresponding to the best feasible solution of RealCase2 in the standard HFS scenario.

**Limitation Statement:** We must acknowledge that the current study focuses solely on discrete manufacturing cases (e.g., water meter production as described in the paper). In future work, we plan to extend our research to process industry scenarios, such as chemical or pharmaceutical manufacturing.

Detailed changes are in the revised manuscript with track changes.

Please check.

2) While the model performs well on medium-sized problems, solving larger or more complex instances—especially in multi-scenario, multi-machine environments—could lead to significant computational time, possibly limiting practical application in large-scale industrial settings.

✓ Thanks for the comments.

We sincerely appreciate your insightful comments regarding the computational efficiency of the proposed model. We fully agree that computational timeliness is critical, especially in large-scale and complex industrial scenarios.

First, as analyzed in Section 5.2, we evaluated the performance of IPMMPO-CP on 40 large-scale benchmark instances. As shown in Table 6, the instance *j30c5e10* required 1050 seconds, which is indeed relatively high for real-time industrial scheduling. Interestingly, however, the instance *j60c5e9* in Table 9 only took 2.6 seconds to solve (we have selected *j60c5e9* as the demonstration case in the revised Supporting Information file). This indicates that problem complexity is not solely determined by instance size, but may also be influenced by constraint satisfaction difficulty. The average runtime of the 30 instances in Table 9 is only 44 seconds. The running results and running time of instance *j60e5e9* are shown in the figure below.

```
! ----------------------------------------------------------------------------
! Search completed, 17 solutions found.
! Best objective         : 1,156 (optimal - effective tol. is 0)
! Best bound             : 1,156
! ----------------------------------------------------------------------------
! Number of branches     : 40,162
! Number of fails        : 5,749
! Total memory usage     : 37.5 MB (36.2 MB CP Optimizer + 1.3 MB Concert)
! Time spent in solve    : 2.40s (2.36s engine + 0.04s extraction)
! Search speed (br. / s) : 17,010.6
! ----------------------------------------------------------------------------
```

In addition, the real-world industrial cases presented in Section 6 show that *RealCase2* required 384.75 seconds under the standard HFS scenario (see Table 11), and 487.36 seconds under the no-wait HFS scenario (see Table 12). Given that production scheduling in the water meter industry is planned on a daily basis, we believe that minute-level computation times are acceptable in such contexts.

Lastly, we would like to emphasize that all experiments in this study were conducted on a relatively modest configuration (Intel i5 CPU with 16GB RAM). When deployed on industrial-grade servers, significant performance improvements can be expected. Accordingly, we have added the following remark in the revised Conclusions section:

[Add point (4) to the revised Conclusions:]

> emission reduction. (4) We fully acknowledge the importance of computational efficiency in solving large-scale, multi-scenario problems. In future work, we plan to integrate the Rolling Horizon Decomposition (RHD) approach to further enhance the timeliness of the proposed method.

Detailed changes are in the revised manuscript with track changes.

Please check.

3) The paper references the design of data libraries and probe libraries essential for the model, but it does not provide detailed insights into their design principles, data completeness, or scalability. Poorly designed libraries could affect model performance and adaptability.

✓ Thanks for the comments. We sincerely appreciate your insightful comments on the design of the data libraries and probe libraries. The detailed design has been presented in Sections 3.1–3.2, and we would like to further clarify the core

principles as follows.

**Decoupled Mapping:** First, the data libraries $X_J$ and $X_M$ strictly follow the binary structures of the conjunctive graph and disjunctive graph, respectively. For instance, data fibers $J_i$ in $X_J$ ensure that the degree of conjunctive arcs for any vertex does not exceed 2, while data fibers $M_j$ in $X_M$ ensure the same constraint for disjunctive arcs. Second, the probe libraries are designed for specific sub-decisions: $Y_S$ is responsible for machine selection within each stage; $Y_M$ handles operation sequencing on the same machine within the same stage; and $Y_J$ ensures inter-stage linkage for the same job, thus preserving technological precedence constraints. To clarify the design principles, we have added the following supplementary explanation at the end of Section 3.2 in the revised manuscript.

**Library Design Principles:**

1. **Isomorphism:** The construction of data libraries is the first and most important. $X_J$ and $X_M$ strictly mirror the binary structures of the conjunctive graph and disjunctive graph, respectively.
2. **Completeness:** For any instance of size $(n, m, s)$, $|X_J| = n \cdot s$, $|X_M| = \sum_{k=1}^{s} n \cdot m_k$ (see Section 3.4 Theorem 1 for details).
3. **Probe-targeted:** The construction of probe libraries is completely dependent on data libraries. Each probe library addresses a specific scheduling sub-decision.

**Data Completeness:** For any instance of size $(n,m,s)$, we have $|X_J| = n \cdot s$ and $|X_M| = \sum_{k=1}^{s} n \cdot m_k$. For example, as shown in Table 2, $|X_J| = 21$ and $|X_M| = 56$. Moreover, there is a one-to-one correspondence between the tuples in $P_{Old,i,k}$ and $X_J$, and between $M_{Old,j,pt}$ and $X_M$, meaning the number of tuples in $P_{Old,i,k}$ and $M_{Old,j,pt}$ exactly matches the number of data types in $X_J$ and $X_M$, respectively.

**Scalability:** According to Theorem 1 in Section 3.4, the space complexity of the data libraries is linear, i.e., **$O(n^2)$**. For example, when scaling up to the large-scale industrial case *RealCase2* (scale: $(50, 22, 6)$), the numbers of tuples in the two data libraries are $|P_{Old,i,k}| = |X_J| = 50 \times 6 = 300$ and $|M_{Old,j,pt}| = |X_M| = 50 \times 22 = 1100$, respectively.

Detailed changes are in the revised manuscript with track changes.

Please check.

4) Although the model shows promising results in simulations and benchmark instances, integrating such a system into actual manufacturing environments might face challenges such as sensor reliability, real-time data collection, and system integration, which are not discussed in the paper.

✓ Thanks for the comments.

Your observation regarding the challenges of industrial deployment is highly insightful, and we fully agree that bridging theory and practice is of critical importance. Although the computational efficiency has been validated through laboratory benchmark testing, we recognize that actual shop-floor deployment requires narrowing the gap between algorithmic innovation and industrial cyber-physical systems. To address this, we have added the following

supplementary statement in the second paragraph of the revised *Conclusions* section.

> **Practical deployment considerations:** While IPMMPO-CP demonstrates computational advantages, its industrial implementation requires: (a) Reliable sensor networks for real-time job tracking (e.g., RFID/IIoT devices); (b) Edge computing infrastructure to handle data collection delays; (c) Middleware integration with existing MES/ERP systems via OPC-UA or API gateways.

Detailed changes are in the revised manuscript with track changes.

Please check.

5) The impact of data uncertainties or parameter variations on the model's solution quality and stability is not explored.

✓ Thanks for the comments.

We appreciate the reviewer's attention to model robustness.

**IPMMPO-CP is a deterministic approach:** As a constraint programming model, all instance inputs (e.g., processing times $pt$ in $M_{Old,j,pt}$) are fixed and known in advance. A key characteristic of deterministic methods is that they always yield a unique optimal strategy once the search terminates, which is fundamentally different from the inherent randomness of heuristic or metaheuristic approximation methods.

**Data uncertainties are beyond scope:** The term 'data uncertainties' refers to stochastic inputs (e.g., probabilistic processing times), which are beyond the scope of our study and violate the problem definition stated in Section 2.1: *"The processing time ... is given in advance."*

**Parameter-free nature:** Unlike heuristic approximation methods such as GA or PSO, the CP-based approach is parameter-free. Input elements such as $n$, $m$, $s$, and $pt$ are deterministic data items, and no algorithmic tuning parameters are required.

Thanks again for your comments.

6) In Table4, it should add "our CP" above the last two columns, such as like Table3. In addition, it's more clear to add "*" for instance (20_20_3) that found by HEA.

✓ Thanks for the comments.

We sincerely thank you for your valuable suggestion regarding the optimization of table presentation. Following your advice, we have made the corresponding revisions and improvements in the updated manuscript. It is worth noting that Table 4 in the original manuscript has been renumbered as Table 5 in the revised version. Since the manuscript was prepared using LaTeX, we have provided the following screenshot for your review.

**Table 5.** Comparison of small- and medium-sized instances from [65].

| Instances | UB | CSA | SA | HEA | our CP | |
|---|---|---|---|---|---|---|
| | | | | | $C_{\max}$ | CPU(s) |
| 10_10_1 | 806 | 794 | 794 | 794 | 794 | **2.7!** |
| 10_10_2 | 785 | 785 | 785 | 785 | 785 | **2.4!** |
| 10_10_3 | 755 | 755 | 755 | 755 | 755 | **1.1!** |
| 10_10_4 | 922 | 914 | 914 | 914 | 914 | **1.4!** |
| 10_10_5 | 969 | 957 | 957 | 957 | 957 | **1.5!** |
| 10_10_6 | 1001 | 1001 | 1001 | 1001 | 1001 | **1.1!** |
| 10_10_7 | 947 | 942 | 940 | 938 | 938 | **2.7!** |

**Table 5 – continued from previous page**

| Instances | UB | CSA | SA | HEA | our CP | |
|---|---|---|---|---|---|---|
| | | | | | $C_{\max}$ | CPU(s) |
| 15_10_3 | 853 | 853 | 853 | 853 | 853 | **15!** |
| 15_10_4 | 886 | 879 | 880 | 883 | **875*** | **150!** |
| 15_10_5 | 1087 | 1076 | 1076 | 1084 | 1076 | **11!** |
| 15_10_6 | 1042 | 1042 | 1042 | 1042 | **1038*** | **12!** |
| 15_10_7 | 1020 | 1020 | 1020 | 1020 | 1020 | **1.6!** |
| 15_10_8 | 1011 | 1011 | 1011 | 1011 | 1011 | **7.9!** |

| | | | | | | |
|---|---|---|---|---|---|---|
| 20_15_10 | 967 | 967 | 966 | 961 | **959*** | 485 |
| 20_20_1 | 1332 | 1333 | 1333 | 1326 | **1322*** | 735 |
| 20_20_2 | 1325 | 1324 | 1325 | 1325 | **1320*** | 168 |
| 20_20_3 | 1324 | 1320 | 1321 | **1315*** | 1316 | 213 |
| 20_20_4 | 1580 | 1578 | 1578 | 1577 | **1576*** | 207 |

Continued on next page

**Table 5 – continued from previous page**

| Instances | UB | CSA | SA | HEA | our CP | |
|---|---|---|---|---|---|---|
| | | | | | $C_{\max}$ | CPU(s) |
| 20_20_5 | 1320 | 1315 | 1319 | 1317 | **1307*** | 424 |
| 20_20_6 | 1284 | 1284 | 1282 | 1280 | 1280 | **23!** |
| 20_20_7 | 1632 | 1630 | 1632 | 1627 | **1623*** | 425 |

Detailed changes are in the revised manuscript with track changes.

Please check.

As required by the editorial office, we have submitted both the *Revised Manuscript with Track Changes* and the clean version *Manuscript* in the revised submission. Please check our revision and reconsider our paper again.

Once again, we appreciate for your warm work, and would like to extend our thanks for the valuable comments.

Looking forward to receiving your approval. Many thanks.

Best regards,

Yours sincerely,

Xiang Tian,

School of Health Management, Binzhou Medical University, Yantai, China

Email: tianxiang08@163.com, (Corresponding author)

Cell Phone: +86-13127086687
